# Vitamin D Determinants, Status, and Antioxidant/Anti-inflammatory-Related Effects in Cardiovascular Risk and Disease: Not the Last Word in the Controversy

**DOI:** 10.3390/antiox12040948

**Published:** 2023-04-18

**Authors:** Giulia Della Nera, Laura Sabatino, Melania Gaggini, Francesca Gorini, Cristina Vassalle

**Affiliations:** 1 Fondazione CNR-Regione Toscana G Monasterio, 56124 Pisa, Italy; 2 Istituto di Fisiologia Clinica, CNR, 56124 Pisa, Italy

**Keywords:** vitamin D, 25(OH)D, antioxidant, determinants, vitamin D status, cardiovascular risk, cardiovascular disease, blood reference levels, threshold, oxidative stress, inflammation, biomarkers

## Abstract

Beyond its key role in calcium homeostasis, vitamin D has been found to significantly affect the cardiovascular (CV) system. In fact, low vitamin D levels have been associated with increased CV risk, as well as increased CV morbidity and mortality. The majority of effects of this molecule are related directly or indirectly to its antioxidative and anti-inflammatory properties. Generally, vitamin D insufficiency is considered for 25-hydroxyvitamin D (25(OH)D) levels between 21–29 ng/mL (corresponding to 52.5–72.5 nmol/L), deficiency as 25(OH)D levels less than 20 ng/mL (<50 nmol/L), and extreme deficiency as 25(OH)D less than 10 ng/mL (<25 nmol/L). However, the definition of an optimal vitamin D status, as defined by 25(OH)D, remains controversial for many extra-bone conditions, including CV disease. In this review, confounding factors affecting the 25(OH)D measurement and status will be discussed. In particular, available evidence on the mechanism and role of vitamin D in relation to CV risk and disease through its antioxidant effect will be reported, also facing the aspect regarding the debate on the minimum blood 25(OH)D level required to ensure optimal CV health.

## 1. Introduction

It is well known that the role of vitamin D extends well beyond the traditional effect on muscle and bone health, now including a number of extra-bone conditions, such as cancer, diabetes, and cardiovascular disease (CVD), many of them related to the antioxidant and anti-inflammatory in which this molecule is involved [1]. Thus, an increase in requests for serum total 25(OH)D, widely recognized as the most reliable marker of vitamin D status, has exponentially grown in the last few years as a consequence of its clinical value, in parallel to the improvement of methodological and standardization-related aspects. However, reference values of vitamin D are still not clearly defined. The Endocrine Society advised reaching serum 25(OH)D levels of at least 30 ng/mL (>75 nmol/L), preferentially to maintain levels in the range of 40–60 ng/mL (100–150 nmol/L) [2]. Accordingly, vitamin D insufficiency is generally considered for 25(OH)D levels between 21–29 ng/mL (corresponding to 52.5–72.5 nmol/L), deficiency as 25(OH)D levels less than 20 ng/mL (<50 nmol/L), and extreme deficiency as 25(OH)D less than 10 ng/mL (<25 nmol/L) [2]. Nonetheless, the Institute of Medicine (IOM) recommended 20 ng/mL as the threshold for physiologically adequate levels of 25(OH)D [3]. In any case, according to these categories, a large percentage of the entire world population can show inadequate serum 25(OH)D levels, especially when considering obese subjects, people with dark skin, and those insufficiently exposed to sunlight, making it necessary to assess the significance of these findings in terms of disease risk [4]. In addition, it has been estimated that a considerable number of children and adolescents are at high risk for vitamin D deficiency and insufficiency worldwide [5].

In this context, it must be said that the threshold of 30 ng/mL has been identified to allow skeletal benefits [6]. At 30 ng/mL or above, vitamin D seems not to limit calcium absorption; levels of parathyroid hormone (PTH; PTH stimulates vitamin D production in the kidney whereas vitamin D acts to inhibit PTH availability) are also minimized at this vitamin D concentration [7,8]. However, in a cross-sectional analysis of more than 300,000 PTH and 25(OH)D samples, no threshold above which increasing 25(OH)D fails to further suppress PTH was observed [9]. Moreover, it has been estimated that the substrate concentration (*K*_m_) of 25(OH)D3 required for 50% maximal activity for the 1alfa-hydroxylase corresponded to 40 ng/mL (100 nmol/L) [10]. However, the levels of this enzyme vary between tissues, and consequently, the requirement of a specific cell type or tissue may differ, so it is likely that blood 25(OH)D concentration may result sufficient, but may fall below critical levels in a particular microenvironment with adverse consequences on the pathophysiology of different extra-skeletal conditions [11]. For these reasons, the issue regarding a threshold, or rather, multiple thresholds, for extra-bone conditions is still debated [11,12]. In particular, although different findings suggest an association between vitamin D status and cardiovascular (CV) risk and disease (e.g., hypertension, diabetes, obesity, coronary artery calcification, stroke, heart disease), and numerous molecular and cellular mechanisms have been hypothesized to explain this relationship (many regarding stress oxidative and inflammatory pathways), it is still undefined which 25(OH)D threshold is more suitable to obtain and maintain CV benefits [13].

In this review, confounding factors affecting the 25(OH)D measurement and status will be discussed. Available evidence on the antioxidant and anti-inflammatory mechanisms and role of vitamin D in relation to CV risk and disease will be also discussed, and an update on the debate regarding the minimum level required to ensure optimal CV health will be reported.

## 2. Vitamin D Metabolism: A Brief Summary

UVB sun rays are the main source of vitamin D, whereas less than 10% derives from dietary intake (e.g., salmon, mackerel and herring, mushrooms, eggs, and fish liver oil), but may be also added to other foods or available as a dietary supplement. Skin exposure to solar UV irradiation induces photolysis of a derivative of cholesterol (7-dehydrocholesterol) into pre-vitamin D3, then isomerized to vitamin D3 (cholecalciferol) [1]. As a liposoluble/hydrophobic molecule, vitamin D3 requires the binding with a transporter protein (vitamin D-binding protein, VDBP) to circulate in the blood. Then, it is hydroxylated in the liver to form 25(OH)D, the major circulating metabolite [1]. In the kidney, hydroxylation catalyzed by the 1alfa-hydroxylase enzyme produces the active hormone, 1,25-dihydroxy vitamin D (1,25(OH)_2_D), while 24-hydroxylase (CYP24) promotes the production of inactive forms [1]. When released by its binding with DBP to the tissues, 1,25(OH)_2_D mediates a number of actions through its intracellular vitamin D receptor (VDR). The main objectives are control of calcium and phosphorus homeostasis (kidney and intestine as principal target tissues) and bone health and turnover. Although 1,25(OH)_2_D represents the active form, there is general agreement on the measure of 25(OH)D as the best index of vitamin D status [2]. Notably, 25(OH)D has a higher concentration in the bloodstream with respect to the active form 1,25(OH)_2_D and shows a longer circulating half-life if compared to 1,25(OH)_2_D (3 weeks vs. 4 h, respectively), thus providing more representative information about the vitamin D status. Moreover, 1,25(OH)_2_D values are regulated through PTH (upregulation) and higher serum calcium and phosphate levels (downregulation). Therefore, because vitamin D deficiency may induce secondary hyperparathyroidism, 1,25(OH)_2_D may result in reduced, normal or even elevated despite evidence of vitamin deficiency.

## 3. Methodological Determinants

### 3.1. Preanalytical Issues

25(OH)D is generally measured in plasma or serum samples, although serum is the most used. In some cases, 25(OH)D levels were found to be higher in heparinized plasma than in serum samples or in ethylenediamine tetraacetic acid (EDTA) plasma [14,15,16]. When tubes with gel are used, no impediment for immunoassay evaluation was registered, whereas interferences have been observed with high-performance liquid chromatography (HPLC) or mass spectrometry approaches [14,17].

Different storage conditions (fresh samples vs. up to 24 h at room temperature, different centrifuging times/temperature, multiple freeze–thaw cycles) did not significantly affect 25(OH)D values [7,14,16,17,18,19]. Long-term stability of 25(OH)D at −20 °C and −80 °C is generally acceptable, although a 15% variation after two months [20] or significant loss after four months at −20 °C was observed [14]. In addition, a variation of 25(OH)D levels after five years of storage also at low temperature (−80 °C) has been reported, which may be taken into consideration for studies involving sample long-term storage [21].

### 3.2. Analytical Issues

There are several different analytical assays available for the determination of 25(OH)D, which include immunoassays (e.g., chemiluminescence immunoassay-CLIA and radioimmunoassay-RIA, high-performance liquid chromatography-HPLC with UV/fluorescence detection, liquid chromatography-mass spectrometry-LC-MS or tandem mass spectrometry-LC-MS/MS) [22,23]. Although LC-MS/MS maintains high analytical performance, aspects related to the high cost of instruments, time-consuming, limited throughput, and complexity of methodological problems requiring skilled professional staff, greatly limit the inclusion of this technique in clinical practice (Table 1). Thus, the introduction of automated immunoassays has enabled rapid uptake of testing and the ability to respond to an ever-increasing demand for vitamin D testing (with the exception of RIA, due to available alternatives that avoid radiolabeled compounds). Nonetheless, these assays still present a highly variable analytical performance, and many cross-reactivity problems with several vitamin D metabolites as well as matrix effects (e.g., heterophilic antibodies) [21] (Table 1).

For many years, different national and international scientific organizations have been working on the Vitamin D Standardization Program (VDSP), aiming to align different 25(OH)D measurement approaches, through the development of a standard reference procedure for vitamin D measurement and a certification program for vitamin D standardization. The effort allowed the publication of a list of certified assays satisfying the performance criterion: ±5% mean bias and overall imprecision of <10% over the range of 22–275 nmol/L for 25(OH)D [24,25,26,27,28]. Nonetheless, although the comparability of 25(OH)D has greatly improved, we are still far from real harmonization as evidenced by results obtained in EQA programs and, consequently, the measurement of vitamin D remains difficult [29,30].

We also performed an analysis of results collected in the 2010–2012 cycles by an Italian/French EQA program, evidencing noteworthy within-lab and between-lab variabilities, as well as significant differences between different methodologies [31]. In these EQAS, each participant may assess their own performance by percentage bias of its results from the All-Laboratory Trimmed Mean (ALTM). However, since the ALTM includes results for all participant methods, it may not represent the “true” value and may change over time as technologies evolve, render impossible to evaluate whether assays are under- or over-estimating the true concentrations. 

At present, the development of standard reference materials (SRM) 972 and 972a by the National Institute of Standardization (NIST), and the availability of validated reference measurement procedures (RMP) from the University of Gent and the Centre of Disease Control and Prevention (CDC), established the international standardization of serum and plasma 25(OH)D measurement [32,33,34]. However, the introduction of reference measurement procedures and materials, although contributing to a better harmonization among methods, leaves the same weaknesses [27,31]. A major cause of variability may be related to the different ability to separate 25(OH)D from VDBP, especially in particular conditions (e.g., pregnant women, chronic kidney disease, or in presence of some VDBP polymorphisms), whereas the organic solvents used in LC-MS/MS precipitate all proteins and separate vitamin D metabolites from carriers [35]. In terms of the clinical agreement, some data suggest that even if methods differ from an analytical point of view and are scarcely comparable, they may show concordance in the evaluation of the vitamin D status and correctly classify 25(OH)D categories. On the other hand, other data from different approaches, have come to attribute a sample either to adequate or hypovitaminosis D category, leading to misclassification according to the assay used [34,36]. Thus, in view of the duration of the differences between laboratories, the first practical suggestion would be to identify a reference laboratory to monitor serial samples from the same subject over time.

Interestingly, being vitamin D essentially transported bound to VDBP (85/90%) in a inactivate form, some experts rise the question of whether an adjunctive evaluation of VDPB could be useful for a better assessment of the vitamin D status. In this regard, LC-MS/MS remains the elective analytical method, retaining higher sensitivity and specificity with respect to immunoassays [37]. Moreover, at the moment many different problems hamper the application of this biomarker, the reason why the determination of this analyte is still challenging is that accuracy of available assays is not satisfactory, and standardization of methods is far to be reached [38].

## 4. Environmental Determinants, Lifestyle Habits, and Skin Pigmentation Affecting 25(OH)D Status

A variety of biological and environmental factors can influence vitamin D status in humans [39]. (Table 2). Synthesis of vitamin D in human skin occurs under ultraviolet exposure, thus any factor (e.g., season, latitude, time of day, cloud, ozone) influencing ultraviolet radiation levels, may significantly affect vitamin D production [40,41]. The day length, which is the period between sunrise and sunset, is dependent on latitude and time of the year, thereby peak levels of 25(OH)D are recorded following the summer months [42,43].

Thus, due to seasonal changes based on sunlight exposure, it would be preferable to evaluate the annual variation to adequately estimate the vitamin D status in each subject [14,44,45,46,47]. Indeed, approximately 85% of the world population lives at latitudes between the 40th parallel north and south and, consequently, these individuals are routinely exposed to sunlight [48]. The remainder population (15%) lives at higher latitudes, receiving relatively lower amounts of sunshine, and in late winter/early spring their vitamin D status typically declines and reaches its nadir [49,50,51].

Consistently, an Italian study showed that levels of 25(OH)D were significantly higher in samples obtained in September/October with respect to those taken from February/March [14]. However, the duration and intensity of exposure to sunlight remain difficult to estimate [49], and even at latitudes such as in Italy, healthy young women, particularly those living in the south of the country, frequently showed vitamin D deficiency (defined as a concentration of <50 nmol/L–20 ng/mL/) [52,53]. Moreover, a systematic review including 200 studies from 46 countries published between 1990 and 2010 reported that the inhabitants of Northern Europe had higher values of 25(OH)D (50–75 nmol/L) than those of Southern Europe, such as Italy, evidencing that living in a “sunny” country may be important but not sufficient to guarantee adequate levels of 25(OH)D [54]. In agreement with these findings, a great percentage of subjects living in Tuscany (Italy) suffered from 25(OH)D insufficiency or deficiency (88%), as also observed in the general Italian population [14,55]. Vitamin D levels may even be insufficient in subpopulations such as Italian athletes who play sports characterized by intense outdoor training and with a good calcium intake, supporting the hypothesis that there is no direct effect of physical activity on vitamin D metabolism and the same factors that influence vitamin D levels in the population are also valid for athletes [52,56].

A pooled estimate applied to European populations revealed that regardless of age range, ethnic mix and latitude of the populations studied, 13.0% of the total population (55,844 subjects) had serum concentration of 25(OH)D < 30 nmol/L on average in the year, while, considering vitamin D deficiency < 50 nmol/L, the percentage of subjects reached 40.0% [50]. In a recent systematic review including 107 studies published from 1990 onwards, Manios et al. (2018) confirmed the high prevalence of low vitamin D status in Southern Europe and the Eastern Mediterranean regions, with an estimated vitamin D deficiency averaging between 16 and 27%, depending on age group [57]. The habit to consume fortified food (e.g., liquid milk products and margarine) in Northern Europe countries have substantially improved the vitamin D status of the population [58]. This fact, together with dietary habits (e.g., a high intake of fatty fish and cod liver oil) may also explain, at least in part, a vitamin D status generally adequate in Nordic countries in Europe, where sunlight is not strong enough to trigger the synthesis of vitamin D in the skin from October to March [42,47,59,60].

Moreover, low 25(OH)D levels in Southern Europe may be due to increased skin pigmentation [57,61]. In fact, whether at low latitudes skin pigmentation is an evolutionary response to the intense solar UVB, at the same time melanin reduces the synthesis of vitamin D [62]. Data from the National Health and Nutrition Examination Survey (NHANES) 2001–2010, collected on 26,010 adults aged ≥18 years, revealed a prevalence of around 72% of vitamin D deficiency among non-Hispanic blacks, compared to a value of 22% among non-Hispanic whites [53]. On the other hand, the decrease in latitude results in a rightward shift in the distribution of 25(OH)D levels among adults of African descent, thus the 25(OH)D levels in European Americans and Africans living in Africa are comparable, and substantially superior to those of African Americans [62,63]. Of note, racial differences in levels of total 25(OH)D could be also explained by genetic polymorphisms in the VDBP and in other vitamin D-associated genes [41,64,65,66]

Ozone effectively absorbs UVB radiation, particularly at shorter wavelengths as revealed by the prevalence of <75 nmol/L 25(OH)D among postmenopausal women, which was much higher in urban residents (84%) compared to rural residents [67]. Interestingly, calculations based on ozone projections provided by the climate model showed that the increase of total ozone content levels in middle and high-latitude regions during this century will result in a reduction in vitamin D synthesis dose by up to 39% [68]. Atmospheric aerosols and clouds generally attenuate surface UVB radiation, with completely overcast clouds attenuating even up to 99% of UVB rays [41,69]. In this context, postmenopausal women living in a suburban district of Shanghai, more exposed to sunshine hours than the downtown area and more employed in agriculture and thus in outdoor activities, exhibited serum 25(OH)D level over 20 ng/mL in 60% of the participants, which confirms the great influence of sunshine exposure on vitamin D status [70].

The use of sunscreens may impair vitamin D synthesis if used in the recommended amount of 2 mg/cm^2^, but not in lesser thickness below 1.5 mg/cm^2^ [71]. Hence, while the next generation of sunscreens will be likely studied with a better benefit-risk ratio in terms of skin cancer prevention and less impairment of vitamin D synthesis, some clothing styles (e.g., Muslim style clothing) are significantly associated with reduced levels of vitamin D [54,72].

## 5. Anthropometric Characteristics

Differences in age together with other factors, including vitamin D receptor gene polymorphisms, and constitutive skin pigmentation are responsible for a not negligible part (up to 15%) of the interpersonal variation in the UVB-induced 25(OH)D synthesis in the skin [73]. It is known that the ability to produce vitamin D3 is impaired in the elderly, with an aging-related progressive reduction in skin levels of 7-dehydrocholesterol [55]. This effect can be further exacerbated by reduced nutritional intake of vitamin D, increasing adiposity, less exposure to sunlight due to immobility, and staying indoors, all common factors in adult aging [55,74]. Additionally, as the kidney plays a central role in the regulation of vitamin D metabolism and circulating levels, a reduced renal function can lead to the inhibition of renal 1α-hydroxylase expression, upregulation of 24-hydroxylase and 1,25(OH2)D degradation, and the resulting vitamin D deficiency observed among patients with chronic kidney disease or undergoing dialysis [75,76]. Vitamin D status could also vary according to sex, although there is contrasting evidence as regards this association [77]. In this context, Muscogiuri et al. (2019) found that 25(OH)D levels were lower in females than males across all body mass index (BMI) categories [77]. Previously, among patients undergoing coronary angiography, females had significantly lower vitamin D levels than males, and the female gender was independently associated with severe vitamin D deficiency [78]. However, a recent study evaluating serum 25(OH)D levels in 21,317 participants reported that the mean levels did not differ by sex, with men having slightly lower levels in winter and higher levels in summer as compared to women [79].

25(OH)D is lipophilic and it has been estimated that about 17% of orally-administered vitamin D dose is stored in adipose tissue and the rest is consumed or metabolized, indicating that adipose tissue acts both as a storage and buffering site of vitamin D [80,81]. Accordingly, clinical studies have found that obese individuals have a greater risk (35–40%) of vitamin D deficiency, regardless of age and latitude [82,83,84]. Vitamin D has been shown to affect adipocyte development, although results from in vitro and in vivo studies assessing the effect of vitamin D in adipogenesis are conflicting [81,85]. Furthermore, vitamin D exerts anti-inflammatory action and most studies have shown that vitamin D decreases inflammation in adipose tissues through cytokine release and adiponectin stimulation [81]. A higher BMI induces lower vitamin D levels but not vice versa, as evidenced in a bidirectional Mendelian randomization analysis to explore the causality and direction of the relationship between BMI and 25(OH)D with the use of genetic markers as instrumental variables [86]. In fact, at present, even vitamin D supplementation can be used to prevent the onset of obesity and associated metabolic disorders, there is no definitive scientific evidence to prove that vitamin D deficiency predisposes to obesity [85].

## 6. Vitamin D and Genetic Determinants

Besides environmental and nutritional aspects, circulating levels of vitamin D are also influenced by genetic patterns. Many investigations on the causal role between vitamin D and several diseased conditions relied on studies on identical and non-identical twin pairs, which normally have similar trait-relevant environments [87] and allow better esteem taking into account genetics and/or environmental effects. These studies have shown that vitamin D concentrations are highly heritable, between 29% and 86% [87,88,89]. In general, the considerable variance in the evaluation of vitamin D heritability depends on many different reasons, such as age, gender, seasonality, and comorbidities [90]. Therefore, well-powered twin studies with reliable controls are fundamental in the determination of genetic contribution to the vitamin D circulating component.

Over time, the studies of genetic determinants associated with the modification of vitamin D circulating levels in humans proceeded through several stages. The first classical approach was the linkage analysis of specific genetic intervals on the chromosome and their relation to the disease [91,92]. In general, linkage analysis conducted on affected subjects of the same family unveils which locus segregates with certain disease/phenotype; therefore, this approach may help to identify which chromosome region(s) may be associated with vitamin D variability in many diseases [91]. However, while linkage analysis is very useful in the identification of genes involved in mendelian disorders, it is not as efficient in the identification of genes involved in quantitative traits, such as vitamin D.

Differently from the linkage analysis-based studies, the candidate gene approach and genome-wide association study (GWAS) allowed the identification of reliable and reproducible associations with vitamin D circulating concentration. By candidate gene studies, it is possible to define if the frequency of a specific variant (single nucleotide polymorphism or gene) is associated with the variation of vitamin D levels, usually in the context of unrelated subjects. Several genes, closely related to vitamin D metabolism, have been studied: CYP2R1 and CYP27B1, involved in vitamin D hydroxylation [93]; GC, encoding for a vitamin D carrier protein [94]; VDR, coding for Vitamin D receptor [94]; CYP24A1, a cytochrome P450 gene [95]. The main issue in candidate gene studies regards the fact that multiple testing correction is not adequately applied and may increase the false-positive rates and the risk of incorrect data interpretation.

GWAS constituted the major advancement in the identification of novel links between specific diseases and their biological determinants. Instead of focusing on a limited number of specific gene variations as in candidate gene studies, this innovative approach, which relies on the haplotype map of the genome and array-based advanced technology, rapidly explores the whole genome. The first GWAS of vitamin D consisted of 1012 related subjects from the Framingham Heart Study and genotyped 70,987 SNPs. However, because of the limited power and coverage of the analysis, none of the SNPs has passed the genome-wide significant p threshold at 5 × 10^−8^, obtained from Bonferroni’s correction for multiple testing [96]. Ultimately, important key findings on vitamin D genetics were obtained though large-scale international efforts to perform GWAS meta-analyses, involving large numbers of individuals from different cohorts, so as to obtain sufficient statistical power to reliably identify an association between circulating vitamin D levels and genetic determinants of several health outcomes [97]. Interestingly, independent GWAS meta-analyses from two different consortiums, resulting from the aggregation of data from an extensive number of cohorts (European ancestry and SUNLIGHT consortium), allowed the identification of strong genome-wide associations with circulating vitamin D at four loci, GC (index SNP:rs2282679), DHCR7/NADSYN1 (rs12785878), CYP2R1 (rs10741657) and CYP24A1 (rs17216707) [97,98,99]. Further expansion of these previous consortiums yielded the identification of some novel loci with significant variants at SEC23A (rs80187220) and AMDHD1 (rs10745742) [97,98,99,100].

In conclusion, together with key environmental factors determining vitamin D levels, genetic aspects may also play a central role. GWAS approach, together with whole-genome sequencing and Mendelian randomization, provides an important breakthrough in the identification of determinants related to vitamin D metabolism with several diseases.

Interestingly, recent data suggested that genetic variations of VDR are associated with changes in metabolic, inflammatory, and oxidative stress parameters in children, providing evidence of how specific VDR polymorphisms may play a role in general susceptibility or protection to cardiometabolic risk and diseases through these clinical biomarkers [101].

## 7. Vitamin D Mechanisms Related to Its Antioxidant/Antiinflammatory Action and Vascular Health

If a causal role of vitamin D for bone health is widely recognized, with vitamin D deficiency associated with most cases of rickets and osteomalacia, a number of genetic, molecular, cellular, and animal studies strongly suggest that vitamin D signaling has many extraskeletal effects, including regulation of cell proliferation, immune and muscle function, skin differentiation, and reproduction, as well as vascular and metabolic properties, which are not strictly related to calcium homeostasis [102,103]. On the other hand, these extraskeletal effects are characterized by some controversies due to conflicting results between observational and interventional studies [104]. Nonetheless, it is well established that vitamin D is able to modulate immune response (Th1/Th2 reduction) [105], while vitamin D deficiency has been associated with numerous conditions (e.g., multiple sclerosis, type 1 diabetes, rheumatoid arthritis, systemic lupus erythematosus, hepatitis, asthma, respiratory infections) and with an increased risk of any type of cancer and a reduced survival rate [104,106].

The biological effects of vitamin D are mediated by VDR, a member of the transcription factor superfamily of nuclear receptors which, upon activation by its binding to the active form of vitamin D and to a retinoid X receptor, translocate to the nucleus where it may regulate the transcription of vitamin D-sensitive target genes within hours or days [107]. Furthermore, VDR localization also at the cell membrane promotes rapid (second to minutes), non-genomic membrane-mediated responses of 1,25(OH)2D3 [108,109]. Of note, although VDR is expressed in virtually all tissues including endothelial cells, and vascular and smooth muscle cells, its presence was initially documented in cardiovascular tissues [110], suggesting a direct role of vitamin D in maintaining cardiovascular function [111]. Besides, epidemiological data indicate a prominent role of vitamin D in cardiovascular health owing to its beneficial effects on vascular endothelial function, blood pressure, and arterial stiffness [112]. In particular, endothelial dysfunction is a key mediator in the development of the atherosclerotic process and, predisposing the vessel to vascular injury, inflammation, vasoconstriction, thrombosis, and, ultimately, plaque rupture, is an important prognostic marker for cardiovascular events [113], but it also occurs in association with several CV risk factors, including hypertension, hypercholesterolemia, and insulin resistance [111,114].

**Figure 1:**
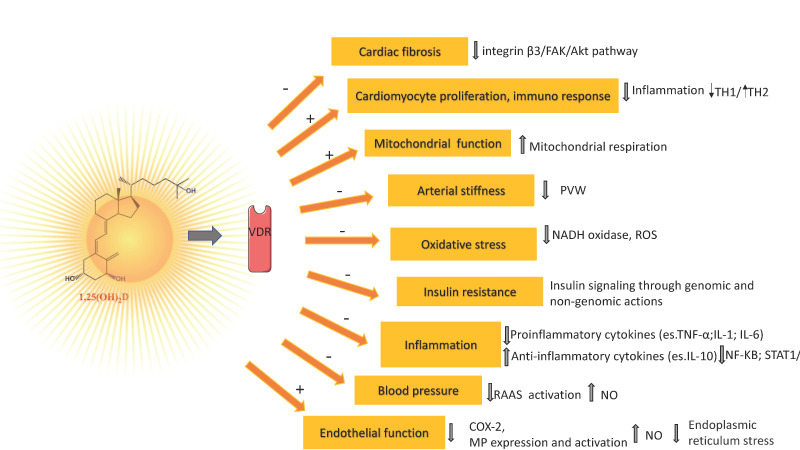
Main Vitamin D mechanisms of actions related to oxidative stress and inflammatory processes and vascular health. ↑ increase, ↓ decrease.

Nitric oxide (NO), produced in the endothelium by endothelial NO synthase (eNOS), in addition to its potent vasodilatory effect, protects the vessels from developing atherosclerosis [115]. Experimental studies reported the ability of vitamin D to stimulate NO production via a direct increase of eNOS gene expression and activation of eNOS in intracellular calcium-dependent pathways (Figure 1) [116,117]. Vitamin D elicits a vasoprotective effect also through a decrease of oxidative stress (a major indicator of NO bioavailability and cause of damage to protein, lipids, and DNA), by upregulating expression of antioxidative enzymes and activating the nuclear factor erythroid 2-related factor 2 antioxidant pathway (Figure 1) [118,119,120].

The chronic inflammation process, which is mediated by several factors, including cell-derived proinflammatory cytokines such as tumor necrosis factor-alpha (TNFα) and interleukin (IL)-1 and IL-6, contributes to the development of endothelial dysfunction, atherosclerosis, and CVD (Figure 1) [115]. The active metabolite of vitamin D has an anti-inflammatory effect through negative regulation of nuclear factor κB (NF-κB) and STAT1/5-mediated signaling, which leads to the downregulation of expression and production of several pro-inflammatory cytokines (TNF-α, IL-1, IL-2β, monocyte chemoattractant protein-1) [121]. Activated VDR also suppresses inflammation through the inhibition of prostaglandin and cyclooxygenase 2 pathways, reduction of matrix metalloproteinase-9, and upregulation of the anti-inflammatory cytokine interleukin (IL)-10 [122].

Renin–angiotensin–aldosterone system (RAAS), a regulatory cascade having a crucial impact on the cardiovascular system tonus through the production of angiotensin II, increases vasoconstriction, extracellular volume, and cardiac output, and represents a major target of vitamin D (Figure 1) [123,124]. In mice, vitamin D was shown to suppress renin transcription by a VDR-mediated mechanism independent of extracellular calcium or phosphorus, which could block the cyclic AMP signaling pathway, a signaling pathway that plays a critical role in renin transcription and release in response to various physiological factors [125,126,127]. The fundamental role of vitamin D in regulating RAAS has been reported in animal models [126,127,128] but, with inconsistent results, in humans [129,130,131]. In fact, although most of the observational studies reported an inverse association between vitamin D and the incidence of hypertension [132,133], pooled results of randomized controlled trials (RCTs) showed that there was no significant reduction in systolic blood pressure or diastolic blood pressure following vitamin D supplementation in the general population [133,134] but may slightly decrease peripheral blood pressure in vitamin D-deficient patients [135]. Notably, the role of vitamin D deficiency in arterial hypertension could be also explained by decreased bioavailability of NO and atherosclerosis, and not exclusively by RAAS hyperactivation [107,117,136]. As for arterial stiffness, a strong predictor of CV events and all-cause mortality, in the meta-analysis of 18 RCTs by Rodriguez et al. (2016), no evidence of significant associations was found between vitamin D supplementation and reductions in pulse wave velocity (PWV) (Figure 1) [137]. Conversely, a recent systematic review and meta-analysis of nine randomized double-blinded placebo-controlled trials, reported that nutritional vitamin D was associated with significant reductions in the pooled difference of the carotid-femoral PWV in vitamin D deficiency populations and the similar result were observed in all sensitivity analyses [138]. Above we reported that low serum 25(OH(D) concentration is linked to a higher BMI. Furthermore, vitamin D deficiency could also cause insulin resistance, interfering with insulin signaling through genomic and non-genomic actions of vitamin D [139]. Thus, the potential link between obesity and insulin resistance, factors that play key roles in the origin of CVD, could be a vitamin D deficiency coexisting with obesity [140,141].

Very recent data confirmed the antifibrotic role of vitamin D, suggesting the downregulation of the integrin β3/FAK/Akt pathway as an underlying mechanism involved in this effect (Figure 1) [142]. Moreover, other experimental data evidenced the effect of calcitriol, which reversed adverse cardiovascular function and cardiac remodeling in post-myocardial infarction mice, suppressing myocardial infarction-induced cardiac inflammation, ameliorating cardiomyocyte death, and promoting cardiomyocyte proliferation (Figure 1) [141]. This evidence may be consequent to VDR upregulation: increased VDR directly interacted with p65, reducing NF-κB signaling and inflammation, moreover, up-regulated VDR translocated into nuclei, bound *IL-10* gene promoter to activate *IL-10* gene transcription, further suppressing inflammation [143].

Vitamin D has a protective effect on vascular endothelial cells by reducing endoplasmic reticulum stress (Figure 1) [144]. Importantly, vitamin D also supports the correct function and activity of the mitochondrial respiratory chain (Figure 1) [145].

## 8. Observational Studies

An inverse relationship between circulating vitamin D levels and different biomarkers related to oxidative stress and inflammation has been found in subjects with cardiometabolic risk or patients with CV disease. In particular, obese subjects (children and adolescents or adults) or T2D patients with hypovitaminosis D presented elevated levels of oxidative stress and inflammatory biomarkers, and an inverse correlation is also found between 25(OH)D and levels of different oxidative stress and inflammatory biomarkers [146,147,148,149,150,151]. In coronary artery disease (CAD) patients, an inverse relationship between vitamin D and homocystine (Hcy) was observed. Moreover, the association of Hcy with CAD severity was significant only among patients with hypovitaminosis D, suggesting that an adequate vitamin D status can prevent the adverse consequences of hyperhomocysteinemia on coronary atherosclerosis [152]. Always in CAD patients, an inverse association between gamma-glutamyltransferase (GGT, another oxidative-related biomarker) and 25(OH)D levels was found [153]. In acute myocardial infarction (AMI), vitamin D was inversely related to metalloproteinases (MMP-2) and leptin, biomarkers known as involved in CAD and AMI [154]. Accordingly, overall, most observational studies have reported an inverse association between vitamin D levels and CVD [103]. In a meta-analysis of 24 prospective studies (22 cohort, two nested case-control; 65,994 participants, of whom 6123 CVD cases; years 1966–February 2012), Wang and co-authors (2012) reported a strong, highly significant, inverse association between low circulating 25(OH)D (range of 20–60 nmol/L) and increased risk of CVD events, estimating a pooled relative risk (RR) of 1.52 (95% confidence interval—95% CI: 1.30–1.77). Similar RRs were estimated for CVD mortality, coronary heart disease, and stroke when the lowest category was compared vs. the highest category of baseline circulating 25(OH)D concentration [155]. In line with these findings, in a meta-analysis of 17 and 16 studies published in 2012, the risk of ischemic heart disease and early death was increased by 39% (25–54%) and 46% (31–64%), respectively, for the lowest vs the highest quartile of 25(OH)D level [156].

In order to assess the relevance of plasma concentrations of 25(OH)D for vascular mortality, a meta-analysis including 12 prospective studies (published up to January 2012) with 4632 vascular deaths, showed that subjects with 25(OH)D in the highest vs. the lowest quarter of distribution, had on average, 21% (95% CI: 13–28%) lower vascular mortality [157]. These results were supported by a following meta-analysis that used data from eight independent prospective cohort studies from Norway, Germany, Iceland, Denmark, and the Netherlands, for a total of 26,916 participants. After adjustment for age, sex, season of blood drawing, BMI, active smoker status, history of CVD, the authors reported that, compared to subjects with 25(OH)D concentrations of 75 to 99.99 nmol/L, the adjusted hazard ratios (HRs) for CV mortality in the 25(OH)D groups with 40 to 49.99, 30 to 39.99, and <30 nmol/L, were 1.65 (95% CI 1.39–1.97), 1.61 (95% CI 1.46–1.77), and 2.21 (1.50–3.26), respectively [158]. A dose-response meta-analysis performed on 13 cohort studies published up to February 2018 and involving 21,079 participants, revealed that among subjects with 25(OH)D level < 50 nmol/L, older adults had a higher mortality of CVD (RR = 1.54 95% CI: 1.24–1.91). In addition, a significantly increased mortality for CVD in older adults was found for the deficient (<25 nmol/L; RR = 1.47, 95% CI: 1.15–1.81) and insufficient (25–50 nmol/L; RR = 1.16, 95% CI 1.04–1.27) categories of 25(OH)D, compared to the reference category of >75 nmol/L [159].

These findings are consistent with the increased likelihood of CVD risk factors in older adults, however, the heterogeneity of measurement assays of vitamin D across studies may represent a non-negligible confounding factor [159]. Conversely, within a previous dose-response meta-analysis of 34 prospective studies for a total of 180,667 participants, Zhang and co-workers found that for 10 ng/mL increment of serum 25(OH)D, pooled RRs were 0.90 (95% CI: 0.86–0.94) and 0.88 (95% CI: 0.80–0.96) for total CVD events and CVD mortality, respectively, although it should be evidenced that the number of participants with high concentrations of serum 25(OH)D was small [160].

The meta-analysis by Zhou et al. (2018), which included 19 studies (15 cohort, three case-control, 1 RCT, published by 2017) aimed at exploring the association between vitamin D and stroke, showed a pooled risk of 1.62 (95% CI: 1.34–1.96). In the subgroup analysis, a lower vitamin D status was associated with ischemic stroke (RR = 2.45, 95% CI: 1.56–3.86), further suggesting a protective role of higher circulating vitamin D against stroke incidence [161]. A meta-analysis including 25 prospective cohort studies (publication years 2000–2017) including more than 10,000 CVD cases, reported that lower levels of vitamin D were associated with an increased RR of CVD (incidence-mortality combined) by 44% and of CVD mortality by 54% [162]. Recently, a population-based retrospective cohort study including a total of 11,002 subjects with a 25(OH)D measurement, showed that the adjusted HRs for new diagnoses of CVD after a median overall follow-up of 4.8 years, were 1.28 (95% CI:1.12–1.46), 1.19 (95% CI: 1.09–1.31), and 1.10 (95% CI: 0.95–1.26) for 25(OH)D values < 12, 12–19, and >50 ng/mL, compared to the reference range 20–50 ng/mL, respectively [162,163]. Hence, no significant association was observed for 25(OH)D values > 50 ng/mL, although it is always important to consider that a single measurement of serum 25(OH)D may not represent long-term vitamin D status [162,163]. In addition, observational studies are susceptible to uncontrolled confounding factors such as outdoor physical activity, dietary habits, and comorbidities, which may always influence serum 25(OH)D levels [164].

Generally, subjects with 25(OH)D blood concentration < 20 ng/mL present a higher CV risk, and frequently a linear trend considering higher 25(OH)D levels vs. CV risk is observed, which strongly suggests that vitamin D exerts beneficial effects on the CV system [165,166,167,168]. Moreover, many observational findings consistently suggest that severe hypovitaminosis D (e.g., <15 ng/mL) is related to excess CV risk. These low levels induce a risk for myocardial infarction with respect to subjects with sufficient 25(OH)D levels (≥30 ng/mL) corresponding to an RR 2.42 (95% CI, 1.35–3.84) in the Health Professionals Follow-up Study (men, age 40–75, free of CVD diagnosis at enrolment in the study) [169]. Additionally, subjects with intermediate 25(OH)D levels (22.6–29.9 ng/mL) showed an increased risk (RR 1.60; 95% CI, 1.10–2.32) compared with those with adequate 25(OH)D. Moreover, in the Framingham Offspring cohort study (n = 1739 participants without prior CVD), subjects with hypertension showed a graded increase in CV risk according to categories of 25(OH)D, with HR 1.53 (95% CI 1–2.36) for levels 10–14 ng/mL and 1.80 (95% CI 1.05–3.08) for levels < 10 ng/mL (*p* = 0.01) respect to those with levels ≥ 15 ng/mL [170].

**Figure 2:**
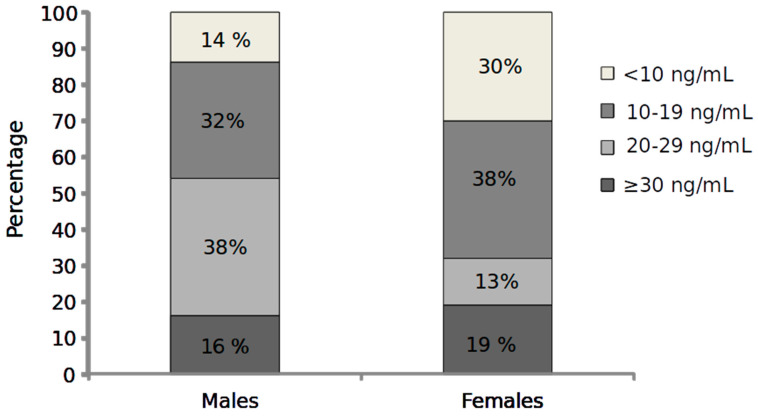
Percentage of Vitamin D categories in male and female AMI patients (*p* < 0.05 χ^2^ test) (reanalyzed data from the cohort of ref. [173].

In a cross-sectional study including 1484 patients undergoing elective coronary angiography, hypovitaminosis D was observed in most subjects. Moreover, Vitamin D deficiency was significantly associated with the prevalence and extent of CAD, and interestingly this relationship was particularly strong in those with values < 10 ng/mL [171]. In the acute field (AMI), the prevalence of vitamin D deficiency is very high, as the prevalence of hypovitaminosis D was present in almost all AMI patients (n = 239), as 75% resulted in vitamin D deficient and 21% were vitamin D insufficient [172]. Unfortunately, these authors did not report the percentage of patients with severe 25(OH)D reduction (<10 ng/mL). In an Italian cohort, we observed that only 16 and 19 % of AMI female and male patients had sufficient 25(OH)D levels, and, although the mean 25(OH) levels were similar in males and females (21 ± 10, and 20 ± 14, ng/mL, respectively), the percentage of those with severe 25(OH)D reduction (<10 ng/mL) resulted in 14 and 30% in males and females, respectively (*p* < 0.05) (Figure 2) [173].

Nonetheless, some studies suggested that for 25(OH)D levels corresponding to 25 ng/mL no further CV beneficial effects are observed [174]. Conversely, other researchers identified in 80 nmol/L (32 ng/mL) the levels associated with the lowest CV and T2D risk [12].

Levels of 25(OH)D have been generally found inversely related to biomarkers of oxidative stress and inflammation in healthy subjects and patients at cardiometabolic risk. In a general population of 452 adults (18–81 yrs) advanced oxidation protein products and advanced glycation end-products associated with fluorescence showed a significant independent association with 25(OH)D_3_ levels [175]. However, in another study, data did not evidence a clear relationship between vitamin D status and oxidative stress biomarkers in a healthy cohort of subjects, although some depleted antioxidant status was observed in those with vitamin D deficiency [176]. Accordingly, in the elderly with impaired glucose metabolism the vitamin D status is inversely associated with levels of circulating markers of oxidative stress (advanced oxidation protein products and low-density lipoprotein susceptibility to oxidation) and endothelial dysfunction, this relationship is particularly significant in subjects with hypovitaminosis D [147]. Levels of IL-6, IL-1β, TNF-α, and Ox-LDL resulted particularly elevated especially in subjects with severe hypovitaminosis D [150]. Interestingly, 25(OH)D levels in children may be predictive of CV risk in adulthood, as shown by the results obtained in a sub-study of the multicenter Cardiovascular Risk in Young Finns Study, showing how in a cohort of 2148 (3–18 years) subjects followed from 1980 to 2007 (30–45 years), children with 25(OH)D levels in the lowest quartile (<40 nmol/L) have a higher risk of carotid atherosclerosis in adulthood, with interesting implications to plan primordial preventive strategies [177].

## 9. Randomized Controlled Trials

Despite extensive evidence suggesting a consistent link between vitamin D and CVD coming from observational studies, overall systematic reviews, and meta-analyses of RCTs did not support any indisputable clear beneficial effect of vitamin D supplementation on CV mortality and risk of total CV events, stroke, and myocardial infarction or ischaemic heart disease, suggesting that vitamin D supplementation does not confer indisputable CV protection. Indeed, Elamin et al. (2012), after having selected 51 eligible studies for a meta-analysis, found no significant impact of vitamin D on MI or stroke and, similarly, on the main CV risk factors (blood lipids, blood glucose, and blood pressure measurements) [178]. In a subsequent meta-analysis evaluating the effects of vitamin D supplementation on extraskeletal outcomes, the authors reported that nutritional vitamin D did not alter the relative risk of any of the cardiovascular endpoints considered—AMI or ischaemic heart disease (nine trials, 48,647 patients) and stroke or cerebrovascular disease (eight trials 46,431 patients)—by 15% or more [179]. In the meta-analysis from Barbarawi et al. (2019) including 21 RCTs for a total of 83,291 participants (of whom 41,669 received vitamin D and 41,622 received placebos), no association was observed between vitamin D supplementation and reduced risk of major adverse cardiovascular events as defined by each trial (primary endpoint), or AMI, stroke, CVD mortality, and all-cause mortality (secondary endpoints) [180]. A more recent systematic review and meta-analysis (18 RCTs published by May 2022, with a total of 70,278 participants eligible for analysis) confirmed the lack of association of vitamin D supplementation with mortality of cardiovascular events as well as with incidence of AMI, stroke, total cardiovascular events or cerebrovascular events [181]. Previously, a Cochrane Library Review concluded that vitamin D supplementation decreased all-cause mortality (75,927 participants; 38 trials), but only when supplemented with vitamin D3 (RR = 0.94 95% CI: 0.91–0.98), whereas vitamin D2, alfacalcidol, or calcitriol had no effects on mortality [182]. Besides, based on the results of the meta-analysis (24 RCTs, 70,528 randomized participants with a median age of 70 years) by Rejnmark et al. (2012), while vitamin D alone did not affect all-cause mortality, the risk of death was significantly reduced during three years of treatment if vitamin D was given with calcium (HR = 0.91 95% CI: 0.84–0.98) [183].

For it concerns the effect of vitamin D supplementation on biomarkers of oxidative stress/inflammation in healthy subjects, it is noteworthy that different biomarker profiles (e.g., total antioxidant capacity-TAC, glutathione, C reactive protein) improve, although for other parameters there is not a clear significant benefit (e.g., malondialdehyde-MDA and carbonyl groups) [184,185,186,187,188]. However, daily intake of vitamin D (150 mg of calcium + 500 IU vitamin D per 250 mL/12 weeks) significantly decreased serum protein carbonyl levels in healthy adults [189]. In T2D hemodialysis patients, vitamin D supplementation induces a significant reduction in hsC-reactive protein and MDA, in parallel to a significant increase in TAC levels [190]. Some data suggested that vitamin D may reduce or prevent the disease progression and cardiovascular risk in T2D patients by decreasing oxidative stress and platelet-mediated inflammation (IL-18, TNF-α, IFN-γ, CXCL-10, CXCL-12, CCL-2, CCL-5, CCL-11, and PF-4), as well as blood vitamin D supplementation (2000 IU/day for six months) in T2D patients having vitamin D < 20 ng/mL resulted associated to a significant decrease in OxLDL, hsCRP, IL-6, PAI-1, and fibrinogen levels and a significant increase in FRAP, (although other studies failed to evidence any significant effect on different biomarkers of oxidative stress and inflammation in these type of patients) [191,192,193,194]. Furthermore, in patients with CAD, there are controversial data on the effects of vitamin D supplementation on oxidative or inflammatory biomarkers related to cardiovascular health [195,196,197]. A recent meta-analysis aiming to assess the effect of vitamin D supplementation on cardiac outcomes in patients with CAD did not evidence any significant effects on hs-CRP mean difference (−0.04, *p* = 0.25), although included a limited number of studies, with small sample size and short duration of interventions [198].

In summary, results from interventional studies, in general, do not support the routine use of vitamin D supplementation, although this strategy could be useful in certain subgroups, where its use may improve metabolic parameters, reducing oxidative stress, inflammation, and CV outcomes [199]. However, it should be noted that the small sample size, the relatively short duration of vitamin D supplementation, and heterogeneity in terms of vitamin D dose, duration of treatment, comorbid conditions, population characteristics, choice of oxidative or inflammatory biomarkers, and assessment of baseline 25(OH)D level across trials could affect the reported results [181].

In addition, other factors, such as type of intervention (only vitamin D vs. vitamin D and calcium), type of vitamin D used (D2 vs. D3), and baseline status (deficiency vs. no deficiency) must be considered [178]. Furthermore, the majority of trials were not designed to evaluate CV outcomes (including CV parameters such as endothelial function, vascular stiffness, or reductions in coronary artery calcium and plaque burden) and were instead performed in populations potentially including many subjects with adequate levels of vitamin D before supplementation (being a baseline 25(OH(D measurement at baseline not performed), thus the controversy over the actual effectiveness of vitamin D supplementation to maintain CV health and prevent adverse CV events remains currently unsolved [122,163,200].

In any case, as current evidence does not definitely establish that vitamin D supplementation confers CV protection, its routinary use for the prevention and treatment of CV disease is not presently advisable in the clinical practice [201,202]. 

Although vitamin D toxicity is a very rare case, it can occur when an excess of supplements is taken (for values > 100 ng/mL–250 nmol/L, not achievable with sunlight exposure or regular food intake, but generally as a consequence of the unintentional assumption of extremely high doses) [203]. In any case, it is important that patients and physicians are conscious that this event can occur, requiring revision of the supplementation (e.g., reduction or cessation) until target 25(OH)D concentration is achieved again [204].

## 10. Conclusions

Data available suggest that 25(OH)D is very stable in serum and that samples do not require any particular caution for transport or storage unless subjected to prolonged storage. Nonetheless, measurement of vitamin D remains difficult for now. Immunoassay remains the most popular method, although presenting different problems which still affect the agreement between methods. Nonetheless, the development of international SRM and the development of reference method procedures are contributing to sharpening the accuracy, precision, and harmonization of results.

UVB exposure has a primary role, but it is also important to consider the contribution of a wide number of environmental, constitutional, life-habit, and genetic determinants able to affect to a large extent the overall vitamin D status. In particular, 25(OH)D is not steady over time. Thus, a single evaluation of 25(OH)D may be insufficient to evaluate the individual vitamin asset, but at least two measurements (e.g., at the nadir-end of winter and zenith-end of summer) are needed.

Hypovitaminosis D is widely spread worldwide. In the CV field, experimental studies confirmed the involvement of this vitamin in CV pathophysiology, the majority related to its antioxidant/anti-inflammatory properties. However, if a major part of the observational trials report an inverse association between vitamin D levels and CV risk and disease and its relationship with different biomarkers of oxidative stress and inflammation in subjects at cardiometabolic risk or with CV disease, randomized controlled studies do not definitively prove that vitamin D supplementation confers CV protection or is able to significantly lower levels of oxidative or inflammatory parameters. It is important to consider how the studies were conducted and whether they are comparable. For example, how much vitamin D the patients took, and for how long? What stage of the disease were they in? It is hard to think that “one size fits all”.

From a clinical point of view, levels for prevention of CV and outcomes (and other extraskeletal conditions) may be likely different from those actually indicated as a normal range (>30 ng/mL) from a bone point of view. This behavior may depend on the fact that different ranges likely reflect different mechanisms or tissue dependence. Thus, it is difficult to precisely define thresholds under or above which no further CV risk can increase or its reduction can be expected, and which patients may benefit from supplementation. Moreover, protecting the condition of blood vessels is a complex network of behaviors involving a correct lifestyle, movement, exercise, and a healthy diet. If a patient developed a CV disease, only taking vitamin D for several weeks can hardly save him. Thus, if there is no need to take hope away from doctors and especially patients regarding the benefit of vitamin D, the definition of its exact role and utility in the clinical CV setting will likely be a long-term process. Future research should address important points still unanswered, together with the improvement of vitamin D analytical issues and standardization, to identify a safe and healthy vitamin D status able to benefit the CV system, so optimized according to the objective and better adapted and tailored in its use for the CV setting, answering to real CV patients’ needs.

## Figures and Tables

**Table 1:** Preanalytical and analytical determinants of 25(OH)D measurement.

Preanalytical Issues	Analytical Issues			
	High-Performance Liquid Chromatography (HPLC), Liquid Chromatography-Mass Spectrometry (LC-MS)		IMMUNOASSAY (Enzyme-Linked Immunosorbent Assay-ELISA, Radioimmunoassay-RIA, Chemiluminescence Immunoassay-CLIA)	
	Advantages	Disadvantages	Advantages	Disadvantages
− very stable in serum − no particular caution for transport or storage, unless prolonged	− high sensitivity and specificity − identification of multiple vitamin D metabolites − analysis in a very wide range of concentration − no matrix effects (e.g., heterophilic antibodies, lipemia, hemolysis)	− specialized instrumentation − complexity of technique − (semi)manual sample preparation − high costs − limited throughput, time-consuming − skilled staff − derivatization site	− possibility of automation − easy of performance, wide diffusion − low volume	− matrix effects − antibody cross-reactivity with − epimers and/or other vitamin D metabolites − interlaboratory variability − between method variability − in the case of RIA, the use of radiolabeled compounds

**Table 2:** Environmental, anthropometric, and lifestyle determinants affecting 25(OH)D levels.

Environmental Determinants	Anthropometric Determinants	Life-Style Determinants
sunlight exposure: intensity and duration season latitude length of day presence of clouds air pollution/ozone	aging race/phototype gender body mass index/obesity genetic asset: presence of specific polymorphisms hepatic/renal dysfunction pregnancy	dietary intake supplementation/fortified foods sunscreen/clothes time spent outdoor/outdoor sports

## Data Availability

Not Applicable.
